# Pioglitazone Inhibits the Expressions of p22^phox^ and p47^phox^ in Rat Mesangial Cells *In Vitro*

**DOI:** 10.1155/2014/601352

**Published:** 2014-02-03

**Authors:** Shan Wang, Shan-dong Ye, Wen-jia Sun, Yuan-yuan Hu

**Affiliations:** Department of Endocrinology, Anhui Provincial Hospital Affiliated to Anhui Medical University, No. 17 Lujiang Road, Hefei 230001, China

## Abstract

*Aim*. The purpose of this study was to investigate the effects of pioglitazone on oxidative stress and the expressions of p22^phox^ and p47^phox^, subunits of NADPH oxidase, in mesangial cells (MCs). *Method*. Rat mesangial cells were cultured and randomly divided into normal glucose (NG) group, high glucose (HG) group, and pioglitazone group. After 48 h exposure, the supernatants and cells were collected. The expressions of p22^phox^ and p47^phox^ in MCs were detected by RT-PCR and western blot. The levels of intracellular ROS were determined by flow cytometry. Coloimetry method was used to detect malondialdehyde (MDA) concentrations and superoxide dismutase (SOD) activities. *Results*. Compared with the NG group, the expression levels of p22^phox^, p47^phox^ and ROS significantly increased, the activity of SOD decreased in HG group, while the concentration of MDA greatly increased (*P* < 0.01). Pioglitazone significantly suppressed HG-induced p22^phox^ and p47^phox^ expressions and oxidative stress. The protein and gene expressions of p22^phox^ and p47^phox^ were markedly reduced after pioglitazone treatment, so did the ROS generation. The activities of SOD in MCs increased, while the concentrations of MDA in the supernatant decreased greatly by pioglitazone. *Conclusions*. Pioglitazone can inhibit HG-induced oxidative stress in MCs through suppressing p22^phox^ and p47^phox^ expressions.

## 1. Introduction

Diabetic nephropathy (DN) is the most common cause of end stage renal failure and is a chronic disease characterized by proteinuria, glomerular hypertrophy, decreased glomerular filtration, and renal fibrosis with loss of renal function. Recent studies have shown that oxidative stress promotes the progression of DN [1, 2]. Nicotinamide adenine dinucleotide phosphate (NADPH) oxidase is the predominant enzyme source for ROS generation, which is composed of five subunits comprising a membrane-associated p22^phox^, a gp91^phox^ subunit and at least four cytosolic subunits: p47^phox^, p67^phox^, p40^phox^, and rac-1/2. Subunits such as p22^phox^ and p47^phox^ were mainly expressed in the kidney. Mesangial cells (MCs), an inherent cell of kidney, play an important role during the development and progression of chronic kidney disease, including DN [3].

Pioglitazone, one of the peroxisome proliferator-activated receptor-*γ*  (PPAR-*γ*) agonists, is used clinically in the treatment of type 2 diabetes through its insulin-sensitizing effect. Accumulating evidences suggest that pioglitazone may be beneficial for DN independent of its hypoglycemic effects [4–6]. The role of pioglitazone in modulating oxidative stress has become appreciated. However, its mechanism of action was still unclear. The present study was undertaken to observe the effects of pioglitazone on expressions of p22^phox^ and p47^phox^ in rat glomerular mesangial cells *in vitro *and explore its potential antioxidative mechanisms.

## 2. Material and Methods 

### 2.1. Reagents

Dulbecco's modified Eagle medium (DMEM) and 1% penicillin/streptomycin were purchased from HyClone (UT, USA); fetal bovine serum (FBS) was purchased from Hangzhou Sijiqing Biological Engineering Materials Co., Ltd. (Hangzhou, China); trypsin, BCA protein assay kit, and the ECL Plus kit were from Beyotime Institute of Biotechnology (Nantong, China); pioglitazone was a gift from Jiang Su Heng Rui Medicine Co., Ltd. (Lianyungang, China); 2′,7′-dichlorofluorescein diacetate (DCFH-DA) and ethidium bromide (EB) were from Sigma (MO, USA); rabbit p22^phox^, p47^phox^ antibody and goat anti-rabbit IgG were purchased from Santa Cruz Biotechnology Inc. (CA, USA); *β*-actin was from Sangon Biological Engineering Technology Corporation (Shanghai, China); TRIzol was obtained from Invitrogen (CA, USA); reverse transcription reagents and pcr kit were from TaKaRa Biotechnology Co., Ltd. (Dalian, China); superoxide dismutase (SOD) and malonaldehyde (MDA) assay were from Nanjing Jiancheng Bioengineering Institute (Nanjing, China).

### 2.2. Cell Culture

MCs (NO. HBZY-1) were purchased from China Center for Type Culture Collection (Wuhan, China). The cells were cultured in DMEM containing 10% FBS and 1% penicillin/streptomycin. Cell culture media were changed every 48–72 h. These cells were grown at 37°C in a humidified 5% CO_2_ incubator and were subcultured at 50–80% confluence using 0.25% trypsin. MCs were plated (2 × 10^5^ cells/well) in six-well plates. After being incubated overnight, the medium was changed to DMEM that contained 1% FBS to render the cells quiescent. An equal number of MCs were randomly divided into three groups as follows: (a) normal glucose (NG): cells were cultured in DMEM containing normal glucose concentration of 5.6 mmol/L and 10% fetal bovine serum, (b) high glucose (HG): cells were maintained in DMEM medium with 10% fetal bovine serum and 25 mM glucose, (c) low-dose pioglitazone group (PIO 1): cells were fed with DMEM medium containing 10% fetal bovine serum, 25 mol/L glucose, and 10^−7^ mol/L pioglitazone, (d) middle-dose pioglitazone group (PIO 2): cells were maintained in DMEM medium with 10% fetal bovine serum, 25 mol/L glucose and 10^−6^ mol/L pioglitazone, and (e) high-dose pioglitazone group (PIO 3): cells were cultured in DMEM medium containing 10% fetal bovine serum, 25 mmol/L glucose, and 10^−5^ mol/L pioglitazone. Pioglitazone was a gift from Jiang Su Heng Rui Medicine Co., Ltd., Lianyungang, China. After 48 h exposure, the supernatants and cells were collected.

### 2.3. RT-PCR for p47^phox^ and p22^phox^ mRNA Expression

Total RNA from cultured cells was extracted using TRIzol reagent according to the instructions. RNA concentration was quantified spectrophotometrically and had a 280/260 optical density ratio between 1.8 and 2.0. After extraction of total RNA, 2 *μ*g was reverse transcribed to cDNA using reverse transcription reagents. The PCR primers were designed by TaKaRa Biotechnology Co., Ltd. (Dalian, China). Primers for p22^phox^, p47^phox^, and glyceraldehyde-3-phosphate dehydrogenase (GAPDH) were synthesized from published sequences as shown in Table 1. PCR cycle for p22^phox^ consisted of denaturing at 94°C for 60 s, annealing at 63.3°C for 60 s, and elongation at 72°C for 60 s, conducted for 30 cycles. When it comes to p47^phox^, the PCR condition was the same as p22^phox^ except for annealing at 58.5°C. The housekeeping GAPDH PCR products were used as an internal control. PCR was performed for 35 cycles of denaturing at 94°C for 30 s, annealing at 52°C for 60 s, and elongation at 72°C for 60 s. All the PCR products were visualized by electrophoresis in 1.5% agarose gel containing 0.5 *μ*g*·*mL^−1^ EB and were analyzed using gel scanner. The ration of p22^phox^/GAPDH and p47^phox^/GAPDH was determined and used to compare groups.

### 2.4. Western Blot

Cells were collected and lysed for western blot. Protein concentrations were measured by using a BCA protein assay kit following the manufacturer's instructions. Equal amounts of protein were loaded by electrophoresis on 10% SDS-PAGE and transferred to PVDF membranes (Bio-Rad Laboratories, USA). Membranes were blocked with 5% fat-free dry milk and were incubated overnight at 4°C with specific antibodies for p22^phox^ (1 : 2000), p47^phox^ (1 : 2000), and *β*-actin (1 : 1000). After being incubated with the respective second antibody (goat antibody against rabbit IgG, 1 : 5000), the ECL Plus kit was used for chemiluminescent detection and captured on X-ray film. Western blot analyses were repeated four times, and qualitatively similar results were obtained.

### 2.5. Detection of Reactive Oxygen Species by Flow Cytometry

Intracelluar reactive oxygen species (ROS) was measured by using the dye probe DCFH-DA according to Shi et al. [7]. This method is based on the oxidation of DCFH-DA by intracellular ROS, resulting in the generation of the highly green fluorescent compound 2′,7′-dichlorofluorescin (DCF), which is polar and trapped within the cells. Cells in different groups were washed with PBS twice and incubated with 20 *μ*mol/L DCFH-DA in the loading medium in 5% CO_2_ at 37°C for 30 min in dark. After DCFH-DA was removed, the cells were washed with PBS and read in fluorescence plate reader at an emission wavelength of 545 nm and an excitation wavelength of 485 nm. Results were expressed as percentage of fluorescence intensity of control.

### 2.6. Measurements of Superoxide Dismutase (SOD) and Malondialdehyde (MDA) in the Supernatant

SOD, acting as the first line of defense against ROS, converts superoxide to H_2_O_2_ [8]. MDA reflects the degree of lipid peroxidation. The activities of SOD and the concentrations of MDA were assayed and calculated with the standard curve according to the manufacturer's instructions.

### 2.7. Statistical Analysis

All results are presented as means ± SD. Differences among groups were evaluated by one-way ANOVA, and the Student-Newman-Keuls test was used for comparison between individual groups. Pearson correlations were adopted to note the correlation. All statistical analyses were performed using SPSS 13.0. The results were considered statistically significant when *P* < 0.05.

## 3. Results

### 3.1. Changes of p22^phox^ and p47^phox^ mRNA Expressions in MCs

**Figure 1:**
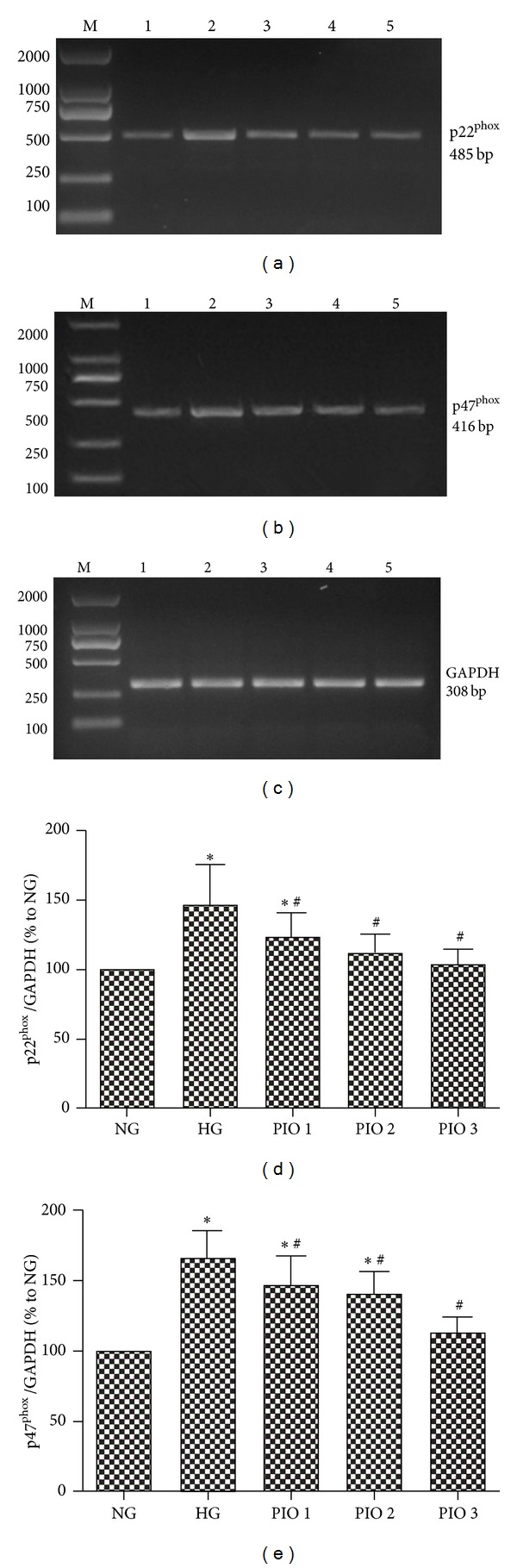
Expressions of p22^phox^ and p47^phox^ mRNA in mesangial cells evaluated by RT-PCR. Gene expressions of p22^phox^ (a), p47^phox^ (b), and GAPDH (c) in mesangial cells. M: maker; 1: NG; 2: HG; 3: PIO 1; 4: PIO 2; 5: PIO 3. Expressions of p22^phox^ (d) and p47^phox^ (e) mRNA among different groups. The values (means ± SD) from six independent experiments are relative to the NG group, which is set as 1.0. *P < 0.01 versus NG group; ^#^ *P* < 0.05 versus HG group.

In this experiment, HG enhanced the p22^phox^ and p47^phox^ mRNA expressions in MCs (Figure 1, *P* < 0.05). However, this upregulation induced by HG was significantly reduced by pioglitazone in a concentration-dependent manner. When treated with pioglitazone, expressions of p22^phox^ mRNA decreased 16.3% in low-dose group (*P* < 0.05), 24.4% in middle-dose group (*P* < 0.05), and 29.8% in high-dose group (*P* < 0.05), while those of p47^phox^ were 11.9%, 14.7%, and 32.2%, respectively.

### 3.2. Changes of p22^phox^ and p47^phox^ Protein Expressions in MCs

**Figure 2:**
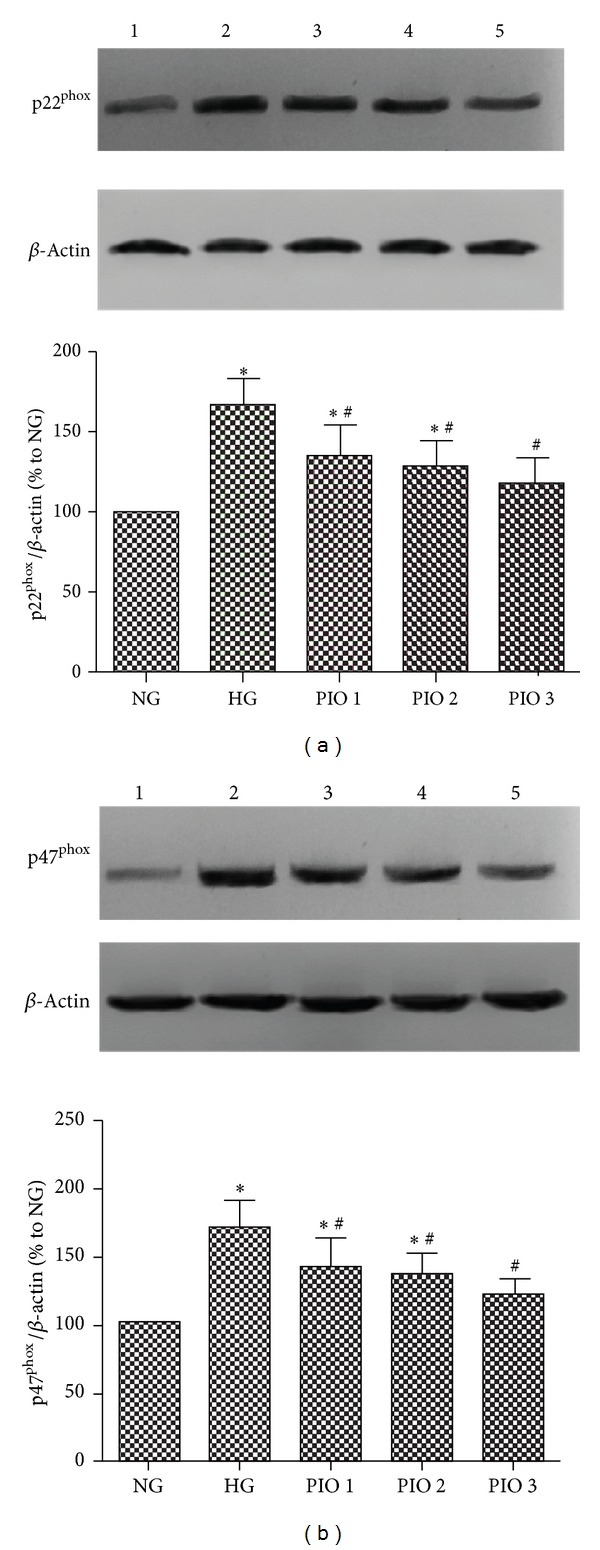
Effects of pioglitazone on protein expression of p22^phox^ and p47^phox^ in HG-treated mesangial cells measured by western blot. (a) Pioglitazone inhibited p22^phox^ expression in mesangial cells. (b) Pioglitazone inhibited p47^phox^ expression in mesangial cells. The values (means ± SD) from six independent experiments are relative to the control, which is set as 1.0. *P < 0.01 versus NG group; ^#^ *P* < 0.05 versus HG group.

MCs treated with HG for 48 h increased p22^phox^ and p47^phox^ protein expression by 1.67-fold and 1.72-fold, respectively (*P* < 0.05). Treatment with different concentrations of pioglitazone (10^−7^, 10^−6^, and 10^−5^ mol/L) markedly inhibited the HG simulated p22^phox^ and p47^phox^ expression in MCs (Figure 2), which had significant differences compared to the HG group (*P* < 0.05).

### 3.3. Intracellular ROS Production in MCs

**Figure 3:**
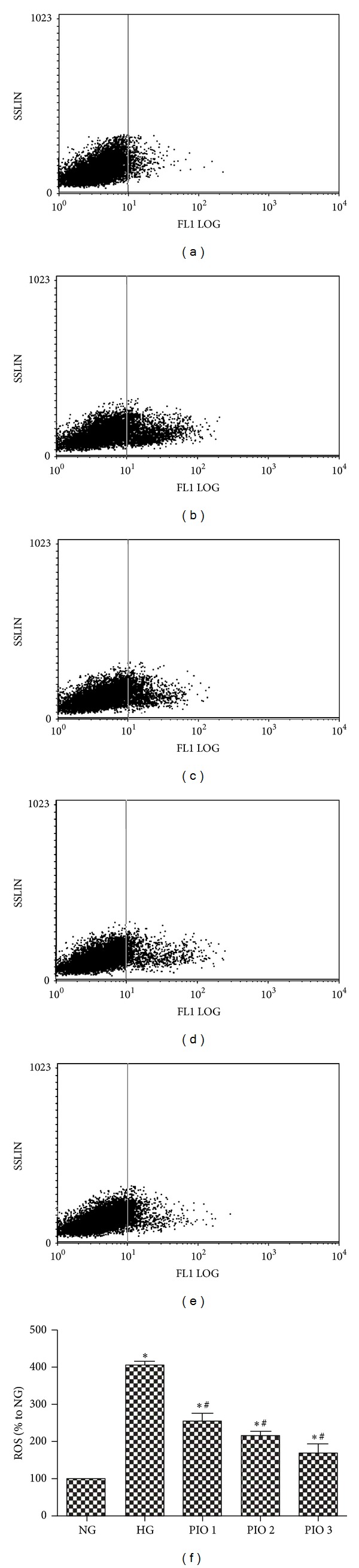
Intracellular ROS production in mesangial cells detected by flow cytometry analysis ((a)–(e)). (a) NG; (b) HG; (c) PIO 1; (d) PIO 2; (e) PIO 3; (f) levels of ROS in mesangial cells among all the groups. The values (means ± SD) from six independent experiments are relative to the NG group, which is set as 1.0. *P < 0.01 versus NG group; ^#^ *P* < 0.05 versus HG group.

To determine whether pioglitazone could induce intracellular ROS generation, levels of ROS production in mesangial cells were determined using the fluorescence probe DCFH-DA. As shown in Figure 3, cells exposed to HG at 25 mmol/L for 48 h displayed a significant increase in the intracellular level of ROS as compared with that in the NG group. When cells were treated with pioglitazone at 10^−7^, 10^−6^, and 10^−5 ^mol/L for 48 h, the intracellular ROS levels decreased significantly compared with those of HG group.

### 3.4. Changes of the SOD Activities and the MDA Concentrations in the Supernatant

**Figure 4:**
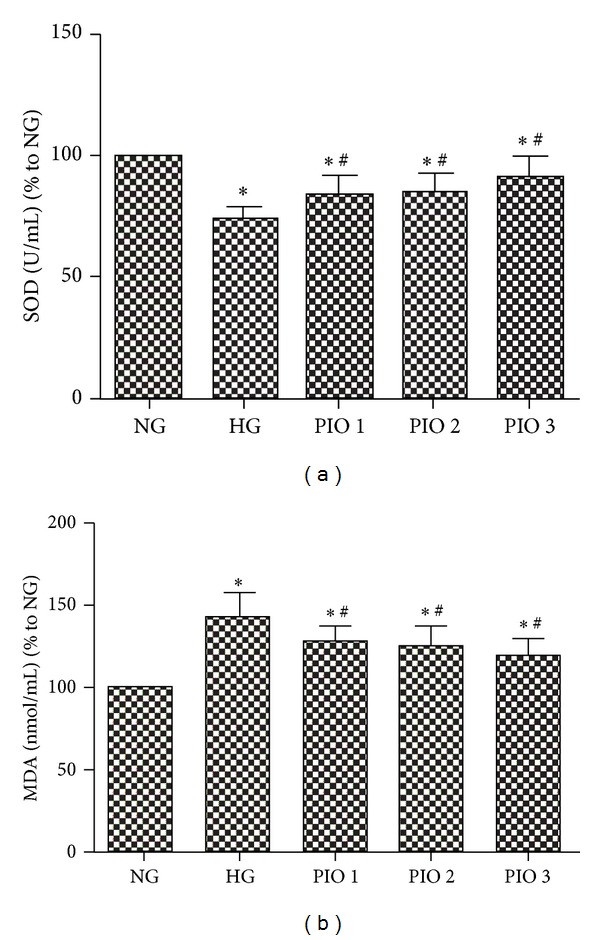
(a) The activities of SOD in the supernatant among different groups. (b) The concentrations of MDA in the supernatant among different groups. The values (means ± SD) from six independent experiments are relative to the NG group, which is set as 1.0. *P < 0.01 versus NG group; ^#^ *P* < 0.05 versus HG group.

Our findings showed a significant decrease in SOD activity and an increase in MDA concentrations in the HG group, compared with the NG group (*P* < 0.01). However, SOD activities were significantly increased and MDA concentrations were markedly decreased after pioglitazone treatment compared with those in the HG group (*P* < 0.05) (Figure 4).

## 4. Discussion

HG is the key factor contributing to long term complications of diabetes mellitus, including DN, which is characterized by mesangial cells proliferation, extracellular matrix accumulation, and tubulointerstitial fibrosis in the glomerular [9], but the molecular mechanisms of chronic complications caused by the exposure to elevated levels of glucose remain to be completely defined. Previous studies have shown that a major link between HG and cellular dysfunction is oxidative stress [10–12].

In diabetes, it has been believed that excessive generation of ROS is the main contributor to lipid peroxidation, oxidation of proteins, and DNA damage, ultimately leading to the development and progression of renal injury [2, 13, 14]. Long-standing hyperglycemia is the hallmark of diabetes and the complications, leading to increasing of ROS production and weakening antioxidant mechanisms [15, 16]. Our study showed that HG significantly increased the generation of ROS in MCs, meanwhile SOD activity in MCs was diminished and MDA concentration in the supernatant increased significantly after 48 h exposure to HG, which indicated that oxidant-antioxidant balance in MC was severely altered under the HG state. NADPH oxidase, a multicomponent enzyme, has been reported to be involved in the generation of ROS under HG condition [17, 18]. In the present study, we detected that the HG significantly improved p22^phox^ and p47^phox^ expressions in MCs, both in mRNA and protein levels, suggesting that activation of NADPH oxidase was possibly responsible for the increased ROS generation in MCs.

The peroxisome proliferator-activated receptor-*γ* (PPAR-*γ*) plays an important role in adipogenesis, glucose metabolism, angiogenesis, and inflammation *in vivo. *Our previous findings demonstrated that pioglitazone can provide a certain renoprotencion for diabetic animals [19] and patients [4], but the underlying mechanisms are less clear. A large body of evidences have demonstrated some roles of pioglitazone in regulating cell proliferation and matrix accumulation by antioxidant induced by HG [20–23]. Jesse et al. [22] showed that pioglitazone markedly protected against the increase in serum urea and creatinine levels and histological alterations in kidney and ameliorated the inhibition of catalase, SOD, and glutathione peroxidase in the kidneys of mice. Yang et al. [23] demonstrated that pioglitazone increases Cu/Zn-SOD and decreases NADPH oxidase protein expression in rats with aging-related progressive renal injury. In agreement with other studies, our result showed that expressions of p22^phox^ and p47^phox^ were increased in MCs under HG condition, as well as intracellular ROS production, which were notably inhibited by pioglitazone with a dose-dependent mode. In addition, decreased SOD activity and increased MDA concentration in MCs induced by HG were also reversed with treatment of pioglitazone, which indicated that pioglitazone plays an antioxidant role against HG-induced oxidative stress by inhibiting p22^phox^ and p47^phox^ expressions in rat mesangial cells.

In summary, our data provide clear evidence that HG can enhance p22^phox^ and p47^phox^ expression, in both gene and protein levels, which were accompanied by the increase of ROS production in rat mesangial cells. Pioglitazone can alleviate HG mediated oxidative stress by inhibiting the p22^phox^ and p47^phox^ expression in rat MCs with a concentration-dependent mode, which may contribute to its reno-protection for diabetic renal injury in some content. However, cells *in vitro* are a model far from real life diabetes. Moreover, many effects of pioglitazone seen *in vitro* are known to be caused by the inhibition of cellular respiration rather than via PPAR-*γ*. The mechanism at work in this case is not clear from the presented experiments and further studies are needed.

## Figures and Tables

**Table 1:** The primer sequences of p22^phox^, p47^phox^, and GAPDH.

	Sequences (5′-3′)	Product size
p22^phox^	F: GAC GCT TCA CGC AGT GGT ACT	485 bp
	R: CAC GAC CTC ATC TGT CAC TGG	
p47^phox^	F: CAG AAT GTT GCC TGG TTG	416 bp
	R: GTG CCC TCC CTT AGA TGA	
GAPDH	F: AGA TCC ACA ACG GAT ACA TT	308 bp
	R: TCC CTC AAG ATT GTC AGC AA	
